# Meta-analysis of the likelihood of FOXC2 expression in early- and late-stage tumors

**DOI:** 10.18632/oncotarget.26087

**Published:** 2018-09-07

**Authors:** Tsutomu Kume, Tarek Shackour

**Affiliations:** ^1^ Feinberg Cardiovascular Research Institute, Northwestern University School of Medicine, Chicago 60611, IL, USA

**Keywords:** FOXC2, cancer, T-Stage

## Abstract

**Background:**

Aberrations in the expression of the transcription factor forkhead box C2 (FOXC2) have been linked to a number of malignancies. Here, we characterized the relationship between FOXC2 and cancer progression by conducting a meta-analysis of studies that reported the frequency of FOXC2 expression in tumors of different stages (T1, T2, T3, T4).

**Methods:**

Relevant articles were retrieved from the Medline database by searching for the terms “FOXC2” and “cancer”; then, the retrieved articles were reviewed individually, and studies that were of multivariate cohort design, evaluated FOXC2 expression via immunohistochemical staining, and assessed the relationship between FOXC2 expression and cancer T-stage were included in our meta-analysis.

**Results:**

Our search terms identified 139 studies, 9 of which met all inclusion criteria. A total of 1433 tumor samples were evaluated in the 9 studies; 596 samples were from early-stage (T1-T2) tumors, and 838 were from late-stage (T3-T4) tumors. FOXC2 was expressed in 46.0% of all samples, in 32.4% of early-stage tumor samples, and in 55.6% of late-stage tumor samples. When calculated relative to early-stage samples, the pooled risk for FOXC2 expression in late-stage samples was 1.367 (95% CI = 1.103–1.695, *p* = 0.004).

**Conclusion:**

The results from our meta-analysis of 9 studies indicate that FOXC2 is 36.7% more likely to be expressed in late-stage tumors than in early-stage tumors.

## INTRODUCTION

Cancer can arise via the accumulation of genetic mutations, which cause the cancer cells to proliferate without restriction, leading to tumor growth and metastasis [1]; however, the molecular and genetic cascades involved in tumor formation and cancer progression are largely unknown. The forkhead box (FOX) family of transcription factors includes 17 subfamilies, from FOXA to FOXR, that control a wide range of biological processes such as cell growth, proliferation, differentiation, and longevity [1]. Notably, aberrant FOXC2 expression has been implicated in a number of malignancies and is correlated with metastasis, chemoresistance, and patient prognosis [1]. For example, FOXC2 regulates epidermal growth factor receptor (EGFR) in glioblastoma, activates two intracellular signaling pathways—mitogen-activated protein kinase (MAPK) and phosphatidylinositol 3-kinase (PI3K)/AKT—that promote the proliferation of colon cancer cells, promotes the proliferation of breast cancer cells by inducing glioma-associated oncogene homolog 1 (GLI1) and sonic hedgehog (SHH)/GLI1 signaling, and accelerates cancer progression by inducing the epithelial mesenchymal transition (EMT). FOXC2 also influences cancer growth and progression by acting as a mediator of both angiogenesis and lymphangiogenesis [1].

The goal of this study was to characterize the relationship between FOXC2 expression and cancer progression by conducting a meta-analysis of studies that reported the frequency of FOXC2 expression in tumors of different stages (T1, T2, T3, T4) [2], and then calculating the pooled relative risk of FOXC2 expression in stage T1-T2 (early) and in stage T3-T4 (late) tumors. We identified 9 reports that met all inclusion criteria and evaluated a total of 1433 samples from a wide range of cancer types. Our results suggest that the frequency of FOXC2 expression is significantly higher in late-stage than in early-stage tumors.

## MATERIALS AND METHODS

Our meta-analysis was conducted and reported according to guidelines provided in the PRISMA for Network Meta-Analyses (PRISMA-NMA) checklist [3]. To reduce the likelihood of duplicate results, we used a single database (Medline), which is consistent with the PRISMA-NMA requirements. Relevant studies were identified by using the PubMed interface to search for the terms “FOXC2” and “cancer,” and the studies included in our meta-analysis were of multivariate cohort design, evaluated FOXC2 expression via immunohistochemical staining, and assessed the relationship between FOXC2 expression and cancer T-stage (T1, T2, T3, T4). The meta-analysis was performed with an open-source program as described previously [4–6].

## RESULTS

### Study selection

**Figure 1:**
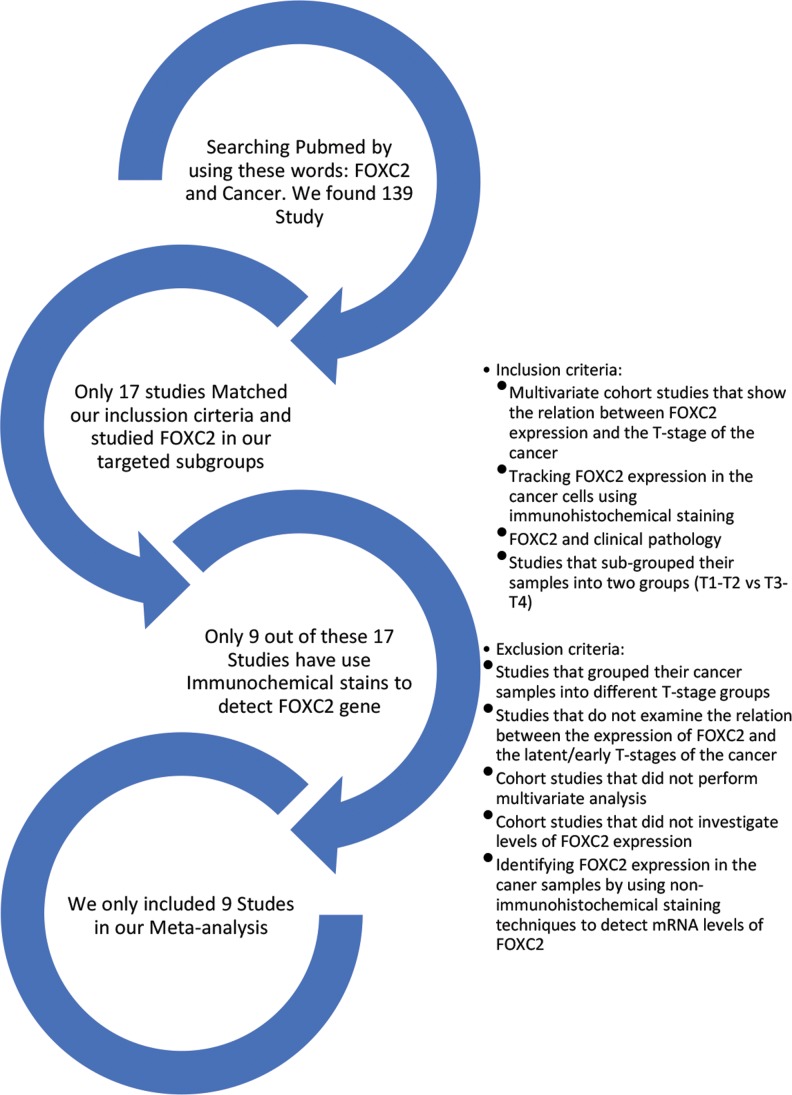
Summary of study selection The Medline database was searched for the terms FOXC2 and Cancer. The studies included in our meta-analysis were multivariate cohort investigations of the relationship between FOXC2 expression and cancer T-stage in which the results were grouped according to tumor stage (T1, T2, T3, T4) and FOXC2 expression was detected via immunohistochemical staining.

The initial Medline search was conducted on and identified 139 articles (Supplementary Table 1); 122 of the studies were excluded because they did not investigate the relationship between FOXC2 expression and cancer or did not categorize their results by tumor stage, and 8 studies did not evaluate FOXC2 expression immunohistochemically (Figure 1). The remaining 9 studies (Table 1) were multivariate analyses and met all inclusion criteria.

**Table 1:** Summary of studies

Study name and location	Year	Type of cancer	T1-T2 Tumor samples			T3-T4 Tumor samples		
			Total	FOXC2^+^	FOXC2^−^	Total	FOXC2^+^	FOXC2^−^
Zhu, *et al*., China	2013	Gastric carcinoma	95	47	44	230	186	95
Lim, *et al*., Singapore	2015	Breast cancer	186	53	67	84	17	186
Sasahira, *et al*., Japan	2014	OSCC	105	26	46	58	12	105
Sun, *et al*., China	2015	RCC	23	13	2	39	37	23
Imayama, *et al*., Japan	2015	OSCC	45	14	8	16	8	45
Cui, *et al*., China	2014	Colorectal cancer	38	16	77	168	91	38
Watanbe, *et al*., Japan	2013	Cholangi carcinoma	40	7	26	37	11	40
Shimoda, *et al*., Japan	2018	Hepatocellular carcinoma	33	8	33	51	18	33
Li, *et al*. China	2015	Colon cancer	30	9	69	155	86	30

OSCC: oral squamous cell carcinoma. RCC: renal cell carcinoma.

### Risk of bias in individual studies

Each individual study had selection bias, because the study samples were not randomly selected. Furthermore, the immunohistochemical methods used to detect FOXC2 expression varied across the studies, and this variation could also induce bias.

### Data collection

#### Zhu, *et al*. (2013) [7]

**Figure 2:**
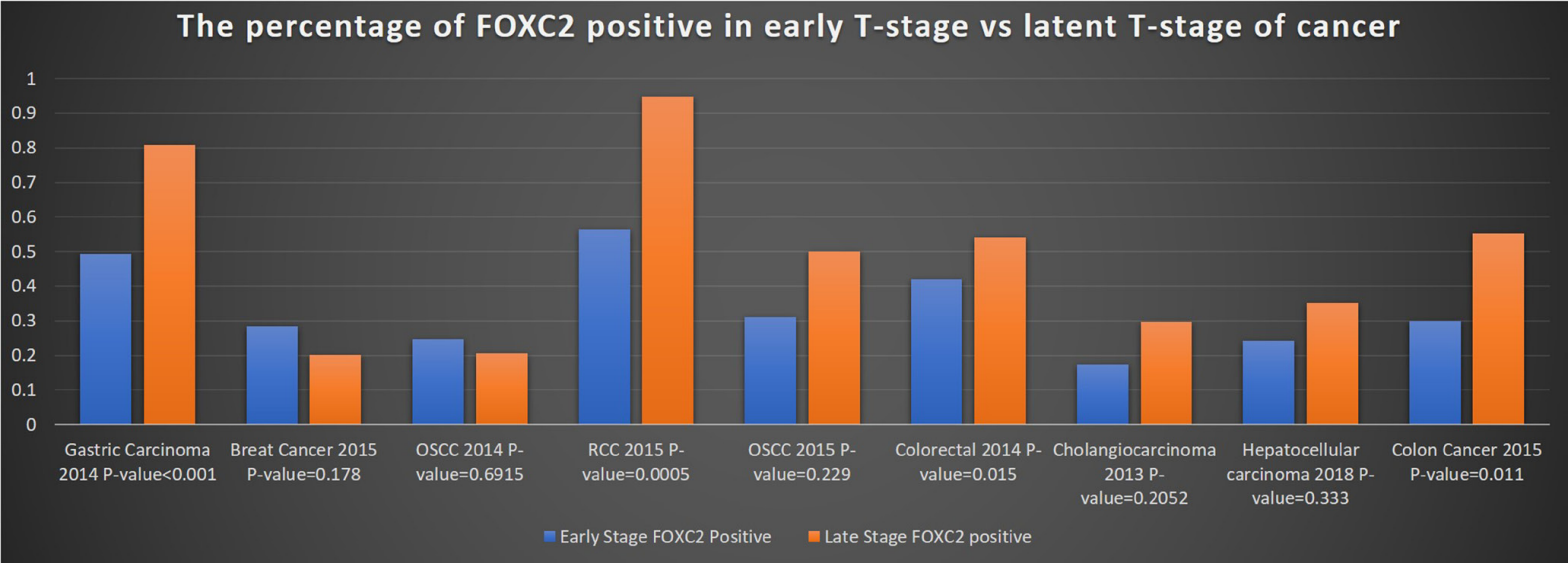
Frequency of FOXC2 expression in early- and late-stage tumors The percentage of early-stage (T1-T2) and late-stage (T3-T4) tumor samples that expressed FOXC2 was calculated for each of the 9 studies included in the meta-analysis and reported according to cancer type. OSCC: oral squamous cell carcinoma; RCC: renal cell carcinoma.

The authors evaluated 325 tumor samples from patients with gastric carcinoma; 95 samples were categorized as stage T1-T2 and 230 samples were categorized as stage T3-T4. High levels of FOXC2 expression were observed in 49.5% (47/95) of T1-T2 samples and in 80.9% (186/230) T3-T4 samples (*p* < 0.001) (Figure 2). For our meta-analysis, “high” expression levels were considered positive for FOXC2 and “low” expression levels were considered FOXC2-negative.

#### Lim, *et al*. (2015) [8]

Samples (*n* = 270) were evaluated from the tumors of patients with breast cancer; 186 samples were from stage T1-T2 tumors, 84 samples were from stage T3-T4 tumors, and the proportion of FOXC2-positive tumors in each group was 28.5% (53/186) and 20.2% (17/84), respectively. The authors found that FOXC2 expression was associated with a higher tumor grade (*p* = 0.178), and that patients with high FOXC2 expression had poorer prognoses and survival.

#### Sasahira, *et al*. (2014) [9]

Samples (*n* = 163) were evaluated from the tumors of patients with oral squamous carcinoma; 31.0% (9/29) of stage T1 tumor samples, 22.4% (17/76) of stage T2 tumor samples, 22.9% (8/35) of stage T3 tumor samples, and 17.4% (4/23) of stage T4 tumor samples were positive for FOXC2 expression. The proportion of FOXC2 positive was not significant (*p* = 0.6915). Data from the T1 and T2 tumors and from the T3 and T4 tumors were combined for our meta-analysis; thus, FOXC2 expression was observed in 24.8% (26/105) of T1-T2 samples and in 20.7% (12/58) of T3-T4 samples.

#### Sun, *et al*. (2015) [10]

Samples (*n* = 62) were evaluated from the tumors of patients with renal cell carcinoma; 80% of cancerous renal tissue samples were positive for FOXC2 expression. Among the 62 tumor samples, the proportion of FOXC2-positive cells was significantly (*p* = 0.0005) for T3-T4 tumors (94.9%; 37 of 39) than for T1-T2 samples (56.5%; 13 of 23).

#### Imayama, *et al*. (2015) [11]

Samples (*n* = 61) were evaluated from the tumors of patients with oral squamous cell carcinoma; 45 of the samples were from T1-T2 tumors, and 16 samples were from T3-T4 tumors. The frequency of FOXC2 expression was 31.1% (14/45) in T1-T2 tumor samples and 50% (8/16) in T3-T4 tumor samples.

#### Cui, *et al*. (2014) [12]

Samples (*n* = 206) were evaluated from the tumors of patients with colorectal cancer. The samples were divided into three groups: stage T1-T2 (*n* = 38), stage T3 (*n* = 110), and stage T4 (*n* = 58). The frequency of FOXC2 expression in the three groups was 42.1% (16/38), 48% (53/110), and 65.5% (38/58), respectively, and differed significantly across the three groups (*p* = 0.015). For our meta-analysis, data from the T3 and T4 tumors were summed, indicating that FOXC2 expression was observed in 54.2% (91/168) of T3-T4 samples.

#### Watanabe, *et al*. (2013) [13]

Samples (*n* = 77) were evaluated from the tumors of patients with cholangiocarcinoma. The samples were categorized as stage T1-T2 (*n* = 40) or stage T3-T4 (*n* = 37), and the frequency of FOXC2 expression in the two groups was 17.5% (7/40) and 29.7% (11/37), respectively.

#### Shimoda, *et al*. (2018) [14]

Samples (*n* = 84) were evaluated from the tumors of patients with hepatocellular carcinoma. Data for each of the four tumor stages were assessed independently; 37.5% (3/8) of stage T1 samples, 20% (5/25) of stage T2 samples, 31.7% (13/41) of stage T3 samples, and 50% (5/10) of stage T4 samples were positive for FOXC2 expression. Thus, when data from the T1 and T2 tumors and from the T3 and T4 tumors were combined for our meta-analysis, FOXC2 expression was observed in 24.2% (8/33) of T1-T2 samples and in 35.3% (18/51) of T3-T4 samples.

#### Li, *et al*. (2015) [15]

Samples (*n* = 185) were evaluated from the tumors of patients with colon cancer. The frequency of FOXC2 expression differed significantly (*p* = 0.011) across the four tumor stages; 0% (0/7) of T1 samples, 39% (9/23) of T2 samples, 50 % (37/73) of T3 samples, and 59% (49/82) of T4 samples were positive for FOXC2 expression. Thus, when summed for our meta-analysis, FOXC2 expression was observed in 30.0% (9/30) of T1-T2 samples and in 55.5% (86/155) of T3-T4 samples.

### Synthesis of results

**Figure 3:**
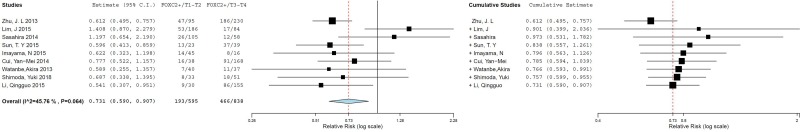
Relative risk of FOXC2 expression in early- and late-stage tumors (individual studies) The relative risk and 95% confidence intervals of FOXC2 expression in early-stage (T1-T2) versus late-stage (T3-T4) and in late-stage versus early-stage tumor samples was calculated for each individual study and displayed in a forest plot.

**Figure 4:**
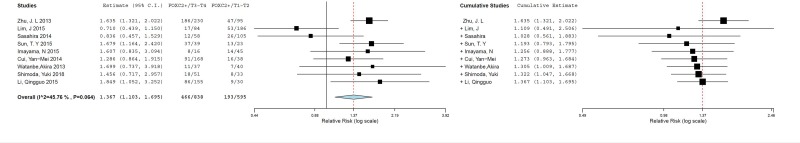
Pooled relative risk of FOXC2 expression in early- and late-stage tumors (meta-analysis of 9 studies) The pooled relative risk and 95% confidence intervals of FOXC2 expression in early-stage (T1-T2) versus late-stage (T3-T4) and in late-stage versus early-stage tumor samples was calculated as the data for all 9 studies was accumulated and displayed in a forest plot.

A total of 1433 tumor samples were evaluated in the 9 studies; 596 samples were from stage T1-T2 tumors, and 838 samples were from stage T3-T4 tumors. FOXC2 was expressed in 46.0% (659/1433) of all samples, in 32.4% (193/596) of T1-T2 samples, and in 55.6% (466/838) of stage T3-T4 samples. Within the individual studies, the relative risk of FOXC2 expression tended to be greater in T3-T4 tumor samples than in T1-T2 tumor samples (Figure 3). Across all nine studies, the pooled relative risk (PRR) for FOXC2 expression in samples from stage T1-T2 tumors versus samples from stage T3-T4 tumors was 0.731 with a 95% confidence interval (95% CI) of 0.590–0.907 and a statistically significant *p*-value (*p* = 0.004) (Figure 4). These results are consistent with those obtained when the pooled relative risk was calculated for stage T3-T4 tumor samples versus stage T1-T2 tumor samples (PRR = 1.367, 95%CI = 1.103–1.695, *p* = 0.004) Thus, the results from our meta-analysis indicate that FOXC2 is 36.7% more likely to be expressed in stage T3-T4 tumors than in stage T1-T2 tumors (Table 2).

**Table 2:** Calculation of pooled relative risk of FOXC2 expression in early- and late-stage tumors (meta-analysis)

Study name	Study weight	Pooled Relative Risk, T1-T2 versus T3-T4					Pooled Relative Risk, T3-T4 versus T1-T2				
		Estimate	95% CI Lower bound	95% CI Upper bound	Standard error	*p*-value	Estimate	95% CI Lower bound	95% CI Upper bound	Standard error	*p*-Value
Zhu, *et al*.	21.4%	0.612	0.495	0.757	0.109	NA	1.635	1.321	2.022	0.109	NA
+ Lim, *et al*.	11.5%	0.901	0.399	2.036	0.416	0.803	1.109	0.491	2.506	0.416	0.803
+ Sasahira, *et al*.	8.6%	0.973	0.531	1.782	0.309	0.929	1.028	0.561	1.883	0.309	0.929
+ Sun, *et al*.	15.2%	0.838	0.557	1.261	0.208	0.398	1.193	0.793	1.795	0.208	0.398
+ Imayama, *et al*.	7.7%	0.796	0.563	1.126	0.177	0.198	1.256	0.888	1.777	0.177	0.198
+ Cui, *et al*.	14.0%	0.785	0.594	1.039	0.143	0.090	1.273	0.963	1.684	0.143	0.090
+Watanbe, *et al*.	5.3%	0.766	0.593	0.991	0.131	0.043	1.305	1.009	1.687	0.131	0.043
+ Shimoda, *et al*.	6.9%	0.757	0.599	0.955	0.119	0.019	1.322	1.047	1.668	0.119	0.019
+ Li, *et al*.	9.4%	0.731	0.590	0.907	0.110	0.004	1.367	1.103	1.695	0.110	0.004
***Final estimate***	100%	0.731	0.590	0.907	0.110	0.004	1.367	1.103	1.695	0.110	0.004

95% CI: 95% confidence interval, NA: not applicable.

### Exploration for inconsistency and risk of bias across studies

Differences in the immunohistochemical protocols and techniques used for FOXC2 staining could produce some inconsistencies in FOXC2 detection. Biases that may affect the cumulative evidence include sample selection, because samples were not chosen randomly in all studies, and our inclusion of multivariate cohort studies which, because they evaluate multiple parameters simultaneously, could increase the heterogeneity of our results. We attempted to minimize heterogeneity by limiting study eligibility. We only selected the multivariate cohort design which evaluated FOXC2 expression via immunohistochemical staining. The I^2^ of the meta-analysis study has shown a moderate heterogeneity with 45.7% with a *p*-value = 0.064 (Table 3).

**Table 3:** Heterogeneity

tau^2^	Q (df = 4)	Heterogeneity *p*-value	I^2^
0.044	14.748	0.064	45.756

## DISCUSSION

Most of the studies chosen in our meta-analysis suggested that FOXC2 expression and cancer were associated, but none of them reported the relative risk between tumor stage and FOXC2 levels. Our meta-analysis compared the expression of FOXC2 in stage T1-T2 and stage T3-T4 tumors, and we reduced selection bias by restricting our analysis to multivariate cohort studies that detected FOXC2 expression via immunohistochemical staining and based the T-stage definition on tumor size, which does not predict morbidity but is a definitive indicator of tumor growth. Our results indicate that FOXC2 expression is significantly more common in later-stage (T3-T4) tumors than in early stage (T1-T2) tumors and, consequently, that FOXC2 may be a marker for the T-stage of cancer.

## SUPPLEMENTARY MATERIALS TABLES




