# Supplementary material for: Meta-analysis of the likelihood of FOXC2 expression in early- and late-stage tumors
Source: Oncotarget. 2018 Sep 7;9(70):33396–402. doi: 10.18632/oncotarget.26087 (PMC6161797; doi:10.18632/oncotarget.26087)
Supplement: Supplementary file 1 [file oncotarget-09-33396-s001.pdf]

Meta-analysis of the likelihood of FOXC2 expression in early- and late-stage tumors

SUPPLEMENTARY MATERIALS

Supplementary Table 1: The Midline search results. See_Supplementary_Table_1
