# Supplementary material for: Meta-analysis of the likelihood of FOXC2 expression in early- and late-stage tumors
Source: Oncotarget. 2018 Sep 7;9(70):33396–402. doi: 10.18632/oncotarget.26087 (PMC6161797; doi:10.18632/oncotarget.26087)
Supplement: Supplementary file 2 [file oncotarget-09-33396-s002.docx]

_Supplementary_Table_1

***Supplementary Table for the Midline search results.***

| **The article titles that did not investigate FOXC2 expression in our targeted group** |
| --- |
| Protein kinase C α enhances migration of breast cancer cells through FOXC2-mediated repression of p120-catenin. |
| NFATC3-PLA2G15 Fusion Transcript Identified by RNA Sequencing Promotes Tumor Invasion and Proliferation in Colorectal Cancer Cell Lines |
| Epithelial‑mesenchymal transition was identified as a potential marker for breast cancer aggressiveness using reverse transcription‑quantitative polymerase chain reaction. |
| Leptin Signaling Mediates Obesity-Associated CSC Enrichment and EMT in Preclinical TNBC Models |
| Identifying biomarkers of breast cancer micrometastatic disease in bone marrow using a patient-derived xenograft mouse model. |
| The Homeotic Protein SIX3 Suppresses Carcinogenesis and Metastasis through Recruiting the LSD1/NuRD(MTA3) Complex. |
| Emerging roles and mechanisms of FOXC2 in cancer |
| Isoform-specific effects of transforming growth factor β on endothelial-to-mesenchymal transition |
| p120-catenin is transcriptionally downregulated by FOXC2 in non-small cell lung cancer cells |
| [Lymphatic vascular system, development and lymph formation. Review]. |
| Whole genome DNA methylation signature of HER2-positive breast cancer. |
| VEGFR-3 controls tip to stalk conversion at vessel fusion sites by reinforcing Notch signalling. |
| Update on the molecular genetics of vascular anomalies. |
| Up-Regulation of FOXC2 and FOXQ1 Is Associated with The Progression of Gastric-Type Adenocarcinoma |
| Transcriptional regulation of WNT2B based on the balance of Hedgehog, Notch, BMP and WNT signals |
| Transcriptional mechanisms of WNT5A based on NF-kappaB, Hedgehog, TGFbeta, and Notch signaling cascades. |
| Transcriptional crosstalk between TGF-β and stem cell pathways in tumor cell invasion: role of EMT promoting Smad complexes. |
| Transcription factors that mediate epithelial-mesenchymal transition lead to multidrug resistance by upregulating ABC transporters. |
| The role of novel prognostic markers PROX1 and FOXC2 in carcinogenesis of oral squamous cell carcinoma. Agnihotri NS1, Astekar M |
| The relationship between gene expression of Forkhead box C2 and tumor progression in cervical carcinoma. Cai L1, Liu D, Lu S, Xu Y, Wang H. |
| The relationship between EMT, CD44high /EGFRlow phenotype, and treatment response in head and neck cancer cell lines. |
| The Role of FoxC2 Transcription Factor in Tumor Angiogenesis. |
| The Foxc2 transcription factor regulates tumor angiogenesis |
| Spry1 and Spry2 are necessary for eyelid closure. |
| Spinal extradural arachnoid cysts in lymphedema-distichiasis syndrome. |
| Spinal extradural arachnoid cysts associated with distichiasis and lymphedema |
| Signalling pathways involved in endocrine resistance in breast cancer and associations with epithelial to mesenchymal transition (Review). |
| Sarcomatoid Urothelial Carcinoma of the Bladder: Analysis of 28 Cases With Emphasis on Clinicopathologic Features and Markers of Epithelial-to-Mesenchymal Transition. |
| Relationship between circulating tumor cells, blood coagulation, and urokinase-plasminogen-activator system in early breast cancer patients. |
| Relationship between circulating tumor cells and epithelial to mesenchymal transition in early breast cancer. |
| Relationship Between Circulating Tumor Cells and Tissue Plasminogen Activator in Patients with Early Breast Cancer. |
| Regulation of the angiopoietin-2 gene by hCG in ovarian cancer cell line OVCAR-3. |
| Protein kinase CK2 in breast cancer: the CK2β regulatory subunit takes center stage in epithelial plasticity. Filhol O1, Giacosa S, Wallez Y, Cochet C. |
| Prostaglandin E2 stimulates β1-integrin expression in hepatocellular carcinoma through the EP1 receptor/PKC/NF-κB pathway. |
| Prognostic value of high FoxC2 expression in resectable non-small cell lung cancer, alone or in combination with E-cadherin expression. |
| Prognostic and diagnostic value of epithelial to mesenchymal transition markers in pulmonary neuroendocrine tumors. |
| Possible genetic predisposition to lymphedema after breast cancer. |
| Phosphorylation of serine 367 of FOXC2 by p38 regulates ZEB1 and breast cancer metastasis, without impacting primary tumor growth. |
| PDT-induced epigenetic changes in the mouse cerebral cortex: a protein microarray study |
| Overexpressed FOXC2 in ovarian cancer enhances the epithelial-to-mesenchymal transition and invasion of ovarian cancer cells. |
| Nuclear localization of GLI1 and elevated expression of FOXC2 in breast cancer is associated with the basal-like phenotype. |
| Notch1 acts via Foxc2 to promote definitive hematopoiesis via effects on hemogenic endothelium. |
| Notch signaling functions in lymphatic valve formation |
| Notch promotes tumor metastasis in a prostate-specific Pten-null mouse model. |
| Molecular mechanisms of lymphatic vascular development. |
| Molecular lymphangiogenesis: new players |
| Molecular characterization of TGFbeta-induced epithelial-mesenchymal transition in normal finite lifespan human mammary epithelial cells |
| Microcephaly, intellectual impairment, bilateral vesicoureteral reflux, distichiasis, and glomuvenous malformations associated with a 16q24.3 contiguous gene deletion and  a Glomulin mutation. |
| MicroRNA Let-7g Directly Targets Forkhead Box C2 (FOXC2) to Modulate Bone Metastasis in Breast Cancer |
| MiR-520h-mediated FOXC2 regulation is critical for inhibition of lung cancer progression by resveratrol |
| Mesenchyme Forkhead 1 (FOXC2) plays a key role in metastasis and is associated with aggressive basal-like breast cancers. |
| Long noncoding RNA expression profiles of the doxorubicin-resistant human osteosarcoma cell line MG63/DXR and its parental cell line MG63 as ascertained by microarray analysis. |
| Lmx1b and FoxC combinatorially regulate podocin expression in podocytes |
| Laminin-binding integrins induce Dll4 expression and Notch signaling in endothelial cells |
| LARP7 suppresses P-TEFb activity to inhibit breast cancer progression and metastasis |
| Isolation and characterization of calcium sensing receptor null cells: a highly malignant and drug resistant phenotype of colon cancer. |
| Interplay of mechanotransduction, FOXC2, connexins, and calcineurin signaling in lymphatic valve formation |
| Integrative genomic analyses on GLI2: mechanism of Hedgehog priming through basal GLI2 expression, and interaction map of stem cell signaling network with P53 |
| Integrative genomic analyses on GLI1: positive regulation of GLI1 by Hedgehog-GLI, TGFbeta-Smads, and RTK-PI3K-AKT signals, and negative regulation of GLI1  by Notch-CSL-HES/HEY, and GPCR-Gs-PKA signals. |
| Integrative genomic analyses of CXCR4: transcriptional regulation of CXCR4 based on TGFbeta, Nodal, Activin signaling and POU5F1, FOXA2, FOXC2, FOXH1, SOX17, and GFI1 t  ranscription factors. |
| Integration-free T cell-derived human induced pluripotent stem cells (iPSCs) from a patient with lymphedema-distichiasis syndrome (LDS) carrying an insertion-deletion complex  mutation in the FOXC2 gene. |
| Inorganic phosphate induces cancer cell mediated angiogenesis dependent on forkhead box protein C2 (FOXC2) regulated osteopontin expression. |
| Inhibition of FOXC2 restores epithelial phenotype and drug sensitivity in prostate cancer cells with stem-cell properties. |
| Identification of HOXD4 Mutations in Spinal Extradural Arachnoid Cyst. |
| Human venous valve disease caused by mutations in FOXC2 and GJC2. |
| Human FOX gene family (Review). |
| Hedgehog target genes: mechanisms of carcinogenesis induced by aberrant hedgehog signaling activation. |
| HGF and MET mutations in primary and secondary lymphedema. |
| Genistein induces apoptosis of colon cancer cells by reversal of epithelial-to-mesenchymal via a Notch1/NF-κB/slug/E-cadherin pathway. |
| Genetic determinants of insulin action in polycystic ovary syndrome. |
| GD3 synthase regulates epithelial-mesenchymal transition and metastasis in breast cancer. |
| Foxc2 transcription factor as a regulator of angiogenesis via induction of integrin beta3 expression |
| Foxc2 induces Wnt4 and Bmp4 expression during muscle regeneration and osteogenesis. |
| Forkhead Box Protein C2 Promotes Epithelial-Mesenchymal Transition, Migration and Invasion in Cisplatin-Resistant Human Ovarian Cancer Cell Line (SKOV3/CDDP). |
| Forkhead Box Protein C2 (FOXC2) Promotes the Resistance of Human Ovarian Cancer Cells to Cisplatin In Vitro and In Vivo. |
| FOXF2 suppresses the FOXC2-mediated epithelial-mesenchymal transition and multidrug resistance of basal-like breast cancer. |
| FOXC2 regulates the G2/M transition of stem cell-rich breast cancer cells and sensitizes them to PLK1 inhibition. |
| FOXC2 promotes colorectal cancer metastasis by directly targeting MET. |
| FOXC2 promotes chemoresistance in nasopharyngeal carcinomas via induction of epithelial mesenchymal transition |
| FOXC2 positively regulates YAP signaling and promotes the glycolysis of nasopharyngeal carcinoma. |
| FOXC2 mutations in familial and sporadic spinal extradural arachnoid cyst |
| FOXC2 is up-regulated in pancreatic ductal adenocarcinoma and promotes the growth and migration of cancer cells. |
| FOXC2 expression links epithelial-mesenchymal transition and stem cell properties in breast cancer. |
| FOXC2 controls formation and maturation of lymphatic collecting vessels through cooperation with NFATc1. |
| FOXC2 augments tumor propagation and metastasis in osteosarcoma. |
| FOXC2 and CLIP4 : a potential biomarker for synchronous metastasis of ≤7-cm clear cell renal cell carcinomas. |
| Expression of transcription factor FOXC2 in cervical cancer and effects of silencing on cervical cancer cell proliferation |
| Expression of epithelial-mesenchymal transition-inducing transcription factors in primary breast cancer: The effect of neoadjuvant therapy |
| Evaluation of cancer stem cell markers CD133, CD44, CD24: association with AKT isoforms and radiation resistance in colon cancer cells. |
| Epithelial-to-mesenchymal and mesenchymal-to-epithelial transitions in the colon. |
| Epithelial-mesenchymal transition-derived cells exhibit multilineage differentiation potential similar to mesenchymal stem cells |
| Epithelial-mesenchymal transition markers in the differential diagnosis of gastroenteropancreatic neuroendocrine tumors. |
| Enhanced expression of retinoic acid receptor alpha (RARA) induces epithelial-to-mesenchymal transition and disruption of mammary acinar structures |
| Elevated FOXC2 Expression Promotes Invasion of HCC Cell Lines and is Associated with Poor Prognosis in Hepatocellular Carcinoma. |
| Effects of forkhead box C2 on carcinogenesis and lymphatic metastasis in endometrial carcinoma |
| Drug-resistant CXCR4-positive cells have the molecular characteristics of EMT in NSCLC |
| Downregulation of Foxc2 enhances apoptosis induced by 5-fluorouracil through activation of MAPK and AKT pathways in colorectal cancer. |
| Distinguishing mechanisms underlying EMT tristability. |
| Disrupting Androgen Receptor Signaling Induces Snail-Mediated Epithelial-Mesenchymal Plasticity in Prostate Cancer. |
| Dietary energy balance modulates epithelial-to-mesenchymal transition and tumor progression in murine claudin-low and basal-like mammary tumor models |
| Defining the E-cadherin repressor interactome in epithelial-mesenchymal transition: the PMC42 model as a case study |
| Defective valves and abnormal mural cell recruitment underlie lymphatic vascular failure in lymphedema distichiasis. |
| Decrypting the PAK4 transcriptome profile in mammary tumor forming cells using Next Generation Sequencing |
| De-SUMOylation of FOXC2 by SENP3 promotes the epithelial-mesenchymal transition in gastric cancer cells |
| Cyclooxygenase-2 induced β1-integrin expression in NSCLC and promoted cell invasion via the EP1/MAPK/E2F-1/FoxC2 signal pathway. |
| Correlation of Forkhead Box c2 with subtypes and invasive ability of invasive breast cancer. |
| Core epithelial-to-mesenchymal transition interactome gene-expression signature is associated with claudin-low and metaplastic breast cancer subtypes. |
| Connexin 47 mutations increase risk for secondary lymphedema following breast cancer treatment. |
| Combining Foxc2 and Connexin37 deletions in mice leads to severe defects in lymphatic vascular growth and remodeling. |
| Claudin 1 expression in basal-like breast cancer is related to patient age |
| Cdk5 controls lymphatic vessel development and function by phosphorylation of Foxc2. |
| Casein kinase 2 prevents mesenchymal transformation by maintaining Foxc2 in the cytoplasm. |
| Canonical Wnt signaling regulates Foxc1/2 expression in P19 cells. |
| Biomedicine and diseases: the Klippel-Trenaunay syndrome, vascular anomalies and vascular morphogenesis. |
| BSTA Promotes mTORC2-Mediated Phosphorylation of Akt1 to Suppress Expression of FoxC2 and Stimulate Adipocyte Differentiation |
| BRCA1 and GATA3 corepress FOXC1 to inhibit the pathogenesis of basal-like breast cancers. |
| Arterial regulators taken up by lymphatics |
| Areca nut contributes to oral malignancy through facilitating the conversion of cancer stem cells. |
| Adipocyte hypoxia promotes epithelial-mesenchymal transition-related gene expression and estrogen receptor-negative phenotype in breast cancer cells |
| A vimentin binding small molecule leads to mitotic disruption in mesenchymal cancers |
| A Forkhead Box Protein C2 Inhibitor: Targeting Epithelial-Mesenchymal Transition and Cancer Metastasis. |
| Prognostic role of Twist, Slug, and Foxc2 expression in stage I non-small-cell lung cancer after curative resection. |

| **The article titles that investigated FOXC2 expression in our subgroup criteria but these studies did not evaluate FOXC2 expression Immunohistochemically.** |
| --- |
| Role and importance of the expression of transcription factor FOXC2 in cervical cancer |
| Long non-coding RNA FOXC2-AS1 predicts poor survival in breast cancer patients and promotes cell proliferation. |
| High expression of forkhead box protein C2 is related to poor prognosis in human gliomas |
| Gene expression of mesenchyme forkhead 1 (FOXC2) significantly correlates with the degree of lymph node metastasis in colorectal cancer. |
| Function and diagnostic value of Anosmin-1 in gastric cancer progression. Kanda M1, Shimizu D1, Fujii T1, Sueoka S1, Tanaka Y1, Ezaka K1, Takami H1, Tanaka H1, Hashimoto R1, Iwata N1, Kobayashi D1, Tanaka C1, Yamada S1, Nakayama G1, Sugimoto H1, Koike M1, Fujiwara M1, Kodera Y1. |
| FOXC2 often overexpressed in glioblastoma enhances proliferation and invasion in glioblastoma cells. |
| FOXC2 is a novel prognostic factor in human esophageal squamous cell carcinoma |
| Antisense lncRNA FOXC2-AS1 promotes doxorubicin resistance in osteosarcoma by increasing the expression of FOXC2. |

| **Our selected multivariate cohort studies that show the relation between FOXC2 expression and the T-stage of the cancer** |
| --- |
| The clinical significance of mesenchyme forkhead 1 (FoxC2) in gastric carcinoma |
| High expression of forkhead box protein C2 is associated with aggressive phenotypes and poor prognosis in clinical hepatocellular carcinoma. |
| Forkhead box protein C2 contributes to invasion and metastasis of extrahepatic cholangiocarcinoma, resulting in a poor prognosis |
| Prognostic significance of epithelial-mesenchymal transition proteins Twist and Foxc2 in phyllodes tumours of the breast |
| Prox1 and FOXC2 Act as Regulators of Lymphangiogenesis and Angiogenesis in Oral Squamous Cell Carcinoma |
| Expression of FOXC2 in renal cell carcinoma and its relationship to clinical pathological features. |
| FOXC2 expression is associated with tumor proliferation and invasion potential in oral tongue squamous cell carcinoma |
| Overexpression of forkhead Box C2 promotes tumor metastasis and indicates poor prognosis in colon cancer via regulating epithelial-mesenchymal transition |
| FOXC2 promotes colorectal cancer proliferation through inhibition of FOXO3a and activation of MAPK and AKT signaling pathways. |
